# Bis[μ-2-(3-pyridylmeth­yl)-2*H*-benzo­triazole]bis­[nitratosilver(I)]

**DOI:** 10.1107/S160053680803479X

**Published:** 2008-10-31

**Authors:** Min Hu, Song-Tao Ma, Liang-Qi Guo, Guang-Hui Sun, Shao-Ming Fang

**Affiliations:** a Zhengzhou University of Light Industry, Henan Provincial Key Laboratory of Surface & Interface Science, Henan, Zhengzhou 450002, People’s Republic of China

## Abstract

In the title centrosymmetric binuclear Ag^I^ complex, [Ag_2_(NO_3_)_2_(C_12_H_10_N_4_)_2_], each Ag^I^ center is coordinated by one pyridine and one benzotriazole N-donor atom of two inversion-related 2-(3-pyridylmeth­yl)-2*H*-benzotriazole (*L*) ligands, and an O atom of a coordinated NO_3_^−^ anion in a distorted T-shaped geometry. This forms a unique box-like cyclic dimer with an intra­molecular non-bonding Ag⋯Ag separation of 6.327 (2) Å. Weak inter­molecular Ag⋯O(nitrate) inter­actions [2.728 (4) and 2.646 (3) Å] link the binuclear units, forming a two-dimensional network parallel to (100). Inter­molecular C—H⋯O hydrogen-bonding inter­actions, involving the *L* ligands and the coordinated NO_3_^−^ anions, link the sheets, forming a three-dimensional framework.

## Related literature

For similar structures, see: Liu *et al.* (2006 ▶, 2007 ▶); Richardson & Steel (2003 ▶); For the synthesis of ligand *L*, see: Liu *et al.* (2008 ▶).
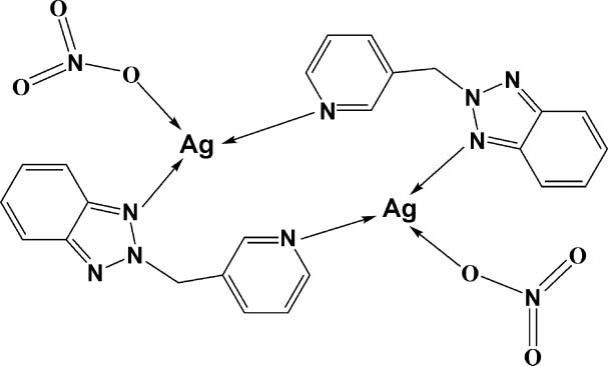


## Experimental

### 

#### Crystal data

[Ag_2_(NO_3_)_2_(C_12_H_10_N_4_)_2_]
*M* *_r_* = 760.24
Monoclinic, 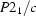

*a* = 10.472 (2) Å
*b* = 8.6921 (17) Å
*c* = 14.656 (3) Å
β = 95.33 (3)°
*V* = 1328.3 (5) Å^3^
*Z* = 2
Mo *K*α radiation
μ = 1.54 mm^−1^
*T* = 293 (2) K
0.20 × 0.15 × 0.11 mm

#### Data collection

Bruker SMART CCD area-detector diffractometer
Absorption correction: multi-scan (*SADABS*; Sheldrick, 2008 ▶) *T*_min_ = 0.749, *T*_max_ = 0.849
12799 measured reflections
2336 independent reflections
2256 reflections with *I* > 2σ(*I*)
*R*_int_ = 0.027

#### Refinement

*R*[*F* ^2^ > 2σ(*F* ^2^)] = 0.036
*wR*(*F* ^2^) = 0.072
*S* = 1.11
2335 reflections
190 parameters
H-atom parameters constrained
Δρ_max_ = 0.96 e Å^−3^
Δρ_min_ = −0.70 e Å^−3^

### 

Data collection: *SMART* (Bruker, 1998 ▶); cell refinement: *SAINT* (Bruker, 1998 ▶); data reduction: *SAINT*; program(s) used to solve structure: *SHELXS97* (Sheldrick, 2008 ▶); program(s) used to refine structure: *SHELXL97* (Sheldrick, 2008 ▶); molecular graphics: *SHELXTL* (Sheldrick, 2008 ▶); software used to prepare material for publication: *SHELXTL* and *PLATON* (Spek, 2003 ▶).

## Supplementary Material

Crystal structure: contains datablocks I, global. DOI: 10.1107/S160053680803479X/su2070sup1.cif

Structure factors: contains datablocks I. DOI: 10.1107/S160053680803479X/su2070Isup2.hkl

Additional supplementary materials:  crystallographic information; 3D view; checkCIF report

## Figures and Tables

**Table:** 

Ag1—N4	2.253 (3)
Ag1—N1^i^	2.311 (3)
Ag1—O3	2.468 (3)
Ag1—O1^ii^	2.728 (4)
Ag1—O2^ii^	2.646 (3)

**Table:** 

N4—Ag1—N1^i^	131.66 (10)
N4—Ag1—O3	127.43 (11)
N1^i^—Ag1—O3	84.66 (11)

Symmetry codes: (i) 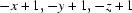; (ii) 
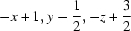
.

**Table 2:** Hydrogen-bond geometry (Å, °)

*D*—H⋯*A*	*D*—H	H⋯*A*	*D*⋯*A*	*D*—H⋯*A*
C5—H5⋯O2^iii^	0.93	2.59	3.365 (3)	141
C6—H61⋯O2^iv^	0.97	2.48	3.416 (5)	161

Symmetry codes: (iii) 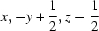; (iv) 
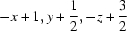
.

**Fig. 1.**
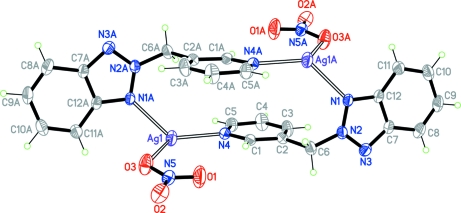
The molecular structure of complex (I). Displacement ellipsoids are drawn at the 30% probability level. Atoms labelled with the suffix A are generated by the symmetry operation (–x + 1, –y + 1, –z + 1).

**Fig. 2.**
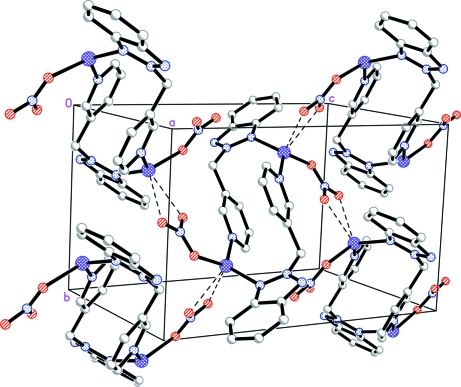
A view of the two-dimensional network of compound (I), parallel to the (100) plane, formed by the intermolecular Ag···O (fine dashed lines) interactions (H atoms have been omitted for clarity).

## supplementary crystallographic information

### Comment

The structures of five N-containing bis-heterocyclic ligands bearing 1-substituted benzotriazole subunits, such as 1-(2-pyridylmethyl)-1*H*-benzotriazole and its binuclear Cu^II^, Pd^II^, and Ag^I^ complexes, have been published previously (Richardson & Steel, 2003). As part of a study on the coordination possibilities of benzotriazole-based ligands with different N-substituted positions in the self-assembly process of coordination complexes, we synthesized a nonplanar flexible ligand based on a 2-substituted benzotriazole subunit and a pendant pyridyl group, namely 2-(3-pyridylmethyl)-2*H*-benzotriazole (*L*). Ligand *L* was then used to construct the title compound, (I), by the reaction of *L* with AgNO_3_.

The structure of compound (I) consists of a centrosymmetric binuclear unit compossed of two *L *ligands, two Ag^I^ centers, and two coordinated NO_3_^-^ anions (Fig. 1). The intramolecular non-bonding Ag···Ag separation is 6.327 (2) Å. Each Ag^I^ center adopts a distorted T-shaped geometry (Table 1) formed by one O atom of a NO_3_^-^ anion and two N-donor atoms; one from the benzotriazole ring system of one *L* ligand, and the other one from the pendant pyridine ring of another *L* ligand.

In this case the 16-membered dimetallocyclic ring is far from planar as a result of the presence of the tetrahedral methylene group of the *L *ligand. All the Ag—O and Ag—N bond distances are in the normal range found for similar complexes (Liu, Chen *et al.*, 2006; Liu, Li *et al.*, 2007).

In the crystal structure adjacent discrete binuclear [Ag(*L*)(NO_3_)]_2_ units are further assembled into one-dimensional chains by intermolecular Ag···O interactions [Ag1···O1^ii^ = 2.728 (4) Å and Ag1···O2^ii ^= 2.646 (3) Å; symmetry code ii: -*x *+ 1, *y* - 1/2, -*z *+ 1.5, see Table 1]. The net result is a two-dimensional network running parallel to the (100) plane (Fig. 2). In addition, the crystal structure of (I) also contains intermolecular C—H···O hydrogen-bonding interactions (Table 2) between the *L* ligands and the coordinated NO_3_^-^ anions that interlink the two-dimensional sheets to form a three-dimensional framework.

We are currently exploring the extension of this study to other 2-substituted benzotriazole-based bis-heterocyclic ligands with bulky aromatic pendant groups, such as acridine and quinoline, and their metal-organic coordination complexes with may have potentially useful properties.

### Experimental

The ligand 2-(3-Pyridylmethyl)-2*H*-benzotriazole (*L*) was synthesized according to the modified method reported in the literature (Liu, Sun *et al.*, 2008). Benzotriazole (0.26 g, 2.2 mmol), 3-(chloromethyl)pyridine hydrochloride (3-picolyl chloride hydrochloride) (0.33 g, 2 mmol), and potassium carbonate (1.52 g, 11 mmol) were added to 50 ml of CH_3_CN. The mixture was stirred at rt for *ca *1 h before being heated at reflux for 24 h, with vigorous stirring. A beige precipitate was obtained, filtered off and rinsed with CH_3_CN. The solvent was removed from the filtrate, and the beige product obtained was taken up in CHCl_3_ and washed three times with H_2_O, before being dried over anhydrous MgSO_4_. Ligand (*L*) was obtained as a yellow powder and purified by recrystallization from CHCl_3_/hexane [Yield: *ca* 40% (based on 3-(chloromethyl)pyridine hydrochloride)]. Elemental analysis calculated for (C_12_H_10_N_4_): C 68.56, H 4.79, N 26.65%; found: C 68.61, H 4.8, N 26.55%. Complex (I) was prepared by adding a solution of AgNO_3_ (0.1 mmol) to a mixture of ligand *L* (0.1 mmol) in CH_3_OH (15 ml) and CH_3_CN (5 ml). A yellow solid formed which was filtered off and the resulting solution was kept at rt. Yellow crystals of complex (I), suitable for X-ray analysis, were obtained by slow evaporation of the solvent after several days. Yield: ~30%. Elemental analysis calculated for (C_12_H_10_AgN_5_O_3_): C 37.92, H 2.65, N 18.42%; found: C 37.81, H 2.70, N 18.34%.

### Refinement

H atoms were included in calculated positions and treated as riding atoms, with C—H = 0.93 (aromatic) or 0.97 Å (methylene), and *U*_iso_(H) = 1.2 or 1.5 *U*_eq_(C). One reflection (100) was omitted from the refinement.

### Crystal data

**Table:**

[Ag_2_(NO_3_)_2_(C_12_H_10_N_4_)_2_]	*F*(000) = 752
*M**_r_* = 760.24	*D*_x_ = 1.901 Mg m^−^^3^
Monoclinic, *P*2_1_/*c*	Mo *K*α radiation, λ = 0.71073 Å
Hall symbol: -P 2ybc	Cell parameters from 4027 reflections
*a* = 10.472 (2) Å	θ = 2.3–28.0°
*b* = 8.6921 (17) Å	µ = 1.54 mm^−^^1^
*c* = 14.656 (3) Å	*T* = 293 K
β = 95.33 (3)°	Block, yellow
*V* = 1328.3 (5) Å^3^	0.20 × 0.15 × 0.11 mm
*Z* = 2	

### Data collection

**Table:**

Bruker SMART CCD area-detector diffractometer	2336 independent reflections
Radiation source: fine-focus sealed tube	2256 reflections with *I* > 2σ(*I*)
graphite	*R*_int_ = 0.027
φ and ω scans	θ_max_ = 25.0°, θ_min_ = 2.0°
Absorption correction: multi-scan (SADABS; Sheldrick, 2008)	*h* = −12→12
*T*_min_ = 0.749, *T*_max_ = 0.849	*k* = −10→10
12799 measured reflections	*l* = −17→17

### Refinement

**Table:**

Refinement on *F*^2^	Primary atom site location: structure-invariant direct methods
Least-squares matrix: full	Secondary atom site location: difference Fourier map
*R*[*F*^2^ > 2σ(*F*^2^)] = 0.036	Hydrogen site location: inferred from neighbouring sites
*wR*(*F*^2^) = 0.072	H-atom parameters constrained
*S* = 1.11	*w* = 1/[σ^2^(*F*_o_^2^) + (0.0212*P*)^2^ + 2.3034*P*] where *P* = (*F*_o_^2^ + 2*F*_c_^2^)/3
2335 reflections	(Δ/σ)_max_ < 0.001
190 parameters	Δρ_max_ = 0.96 e Å^−^^3^
0 restraints	Δρ_min_ = −0.70 e Å^−^^3^

### Special details

**Table:**

Geometry. All e.s.d.'s (except the e.s.d. in the dihedral angle between two l.s. planes) are estimated using the full covariance matrix. The cell e.s.d.'s are taken into account individually in the estimation of e.s.d.'s in distances, angles and torsion angles; correlations between e.s.d.'s in cell parameters are only used when they are defined by crystal symmetry. An approximate (isotropic) treatment of cell e.s.d.'s is used for estimating e.s.d.'s involving l.s. planes.
Refinement. Refinement of *F*^2^ against ALL reflections. The weighted *R*-factor *wR* and goodness of fit *S* are based on *F*^2^, conventional *R*-factors *R* are based on *F*, with *F* set to zero for negative *F*^2^. The threshold expression of *F*^2^ > σ(*F*^2^) is used only for calculating *R*-factors(gt) *etc*. and is not relevant to the choice of reflections for refinement. *R*-factors based on *F*^2^ are statistically about twice as large as those based on *F*, and *R*- factors based on ALL data will be even larger.

### Fractional atomic coordinates and isotropic or equivalent isotropic displacement parameters (Å^2^)

**Table:**

	*x*	*y*	*z*	*U*_iso_*/*U*_eq_	
Ag1	0.44311 (3)	0.21531 (4)	0.62448 (2)	0.05524 (13)	
C1	0.6409 (3)	0.4663 (4)	0.5666 (2)	0.0383 (8)	
H1	0.6092	0.5197	0.6146	0.046*	
C2	0.7353 (3)	0.5350 (4)	0.5209 (2)	0.0377 (7)	
C3	0.7781 (4)	0.4566 (5)	0.4484 (3)	0.0568 (10)	
H3	0.8412	0.4990	0.4155	0.068*	
C4	0.7268 (5)	0.3148 (5)	0.4249 (3)	0.0656 (12)	
H4	0.7537	0.2613	0.3752	0.079*	
C5	0.6361 (4)	0.2536 (4)	0.4753 (3)	0.0527 (10)	
H5	0.6034	0.1566	0.4599	0.063*	
C6	0.7883 (4)	0.6881 (4)	0.5539 (2)	0.0485 (9)	
H61	0.7202	0.7483	0.5769	0.058*	
H62	0.8538	0.6715	0.6043	0.058*	
C7	0.9795 (3)	0.8736 (4)	0.4051 (2)	0.0407 (8)	
C8	1.0899 (4)	0.9289 (5)	0.3673 (3)	0.0568 (10)	
H8	1.1719	0.9070	0.3940	0.068*	
C9	1.0709 (4)	1.0148 (5)	0.2908 (3)	0.0590 (11)	
H9	1.1417	1.0539	0.2646	0.071*	
C10	0.9472 (4)	1.0472 (5)	0.2493 (3)	0.0590 (11)	
H10	0.9391	1.1065	0.1962	0.071*	
C11	0.8387 (4)	0.9950 (5)	0.2840 (2)	0.0505 (9)	
H11	0.7573	1.0167	0.2560	0.061*	
C12	0.8568 (3)	0.9069 (4)	0.3639 (2)	0.0374 (7)	
N1	0.7703 (3)	0.8416 (3)	0.41543 (19)	0.0398 (7)	
N2	0.8434 (3)	0.7743 (3)	0.48214 (19)	0.0405 (7)	
N3	0.9691 (3)	0.7872 (4)	0.4807 (2)	0.0468 (7)	
N4	0.5926 (3)	0.3275 (3)	0.5456 (2)	0.0416 (7)	
N5	0.4289 (3)	0.4142 (4)	0.8019 (2)	0.0487 (8)	
O1	0.5156 (4)	0.4675 (5)	0.7624 (2)	0.0997 (13)	
O2	0.4036 (3)	0.4733 (4)	0.8748 (2)	0.0705 (8)	
O3	0.3683 (3)	0.3011 (4)	0.7710 (2)	0.0759 (10)	

### Atomic displacement parameters (Å^2^)

**Table:**

	*U*^11^	*U*^22^	*U*^33^	*U*^12^	*U*^13^	*U*^23^
Ag1	0.04179 (18)	0.0525 (2)	0.0734 (2)	−0.00689 (13)	0.01612 (14)	0.00621 (15)
C1	0.0340 (17)	0.044 (2)	0.0370 (17)	0.0018 (15)	0.0035 (14)	0.0029 (15)
C2	0.0400 (18)	0.0372 (18)	0.0360 (17)	−0.0006 (15)	0.0036 (14)	0.0048 (15)
C3	0.065 (3)	0.051 (2)	0.058 (2)	−0.007 (2)	0.029 (2)	−0.0018 (19)
C4	0.085 (3)	0.054 (3)	0.063 (3)	−0.008 (2)	0.031 (2)	−0.015 (2)
C5	0.059 (2)	0.040 (2)	0.059 (2)	−0.0071 (18)	0.0048 (19)	−0.0036 (18)
C6	0.060 (2)	0.048 (2)	0.0391 (19)	−0.0129 (18)	0.0110 (17)	0.0021 (17)
C7	0.0407 (19)	0.0393 (19)	0.0431 (19)	−0.0094 (15)	0.0091 (15)	−0.0064 (16)
C8	0.041 (2)	0.066 (3)	0.065 (3)	−0.0151 (19)	0.0127 (18)	−0.010 (2)
C9	0.058 (3)	0.064 (3)	0.059 (3)	−0.025 (2)	0.024 (2)	−0.008 (2)
C10	0.081 (3)	0.052 (2)	0.045 (2)	−0.021 (2)	0.017 (2)	0.0025 (19)
C11	0.054 (2)	0.053 (2)	0.045 (2)	−0.0077 (18)	0.0065 (17)	0.0038 (18)
C12	0.0389 (18)	0.0356 (18)	0.0387 (18)	−0.0084 (14)	0.0088 (14)	−0.0068 (15)
N1	0.0361 (15)	0.0445 (16)	0.0394 (15)	−0.0103 (13)	0.0063 (12)	0.0011 (13)
N2	0.0413 (16)	0.0413 (16)	0.0397 (15)	−0.0092 (13)	0.0086 (13)	0.0008 (13)
N3	0.0400 (17)	0.0509 (18)	0.0495 (18)	−0.0052 (14)	0.0038 (13)	0.0006 (15)
N4	0.0363 (15)	0.0405 (16)	0.0475 (17)	−0.0034 (13)	0.0008 (13)	0.0040 (14)
N5	0.0404 (17)	0.0463 (18)	0.060 (2)	−0.0007 (15)	0.0081 (15)	−0.0028 (16)
O1	0.091 (3)	0.124 (3)	0.089 (2)	−0.053 (2)	0.038 (2)	−0.001 (2)
O2	0.0648 (19)	0.067 (2)	0.081 (2)	−0.0013 (15)	0.0153 (16)	−0.0275 (17)
O3	0.080 (2)	0.071 (2)	0.081 (2)	−0.0322 (18)	0.0301 (17)	−0.0311 (17)

### Geometric parameters (Å, °)

**Table:**

Ag1—N4	2.253 (3)	C7—N3	1.351 (5)
Ag1—N1^i^	2.311 (3)	C7—C12	1.399 (5)
Ag1—O3	2.468 (3)	C7—C8	1.412 (5)
Ag1—O1^ii^	2.728 (4)	C8—C9	1.345 (6)
Ag1—O2^ii^	2.646 (3)	C8—H8	0.9300
C1—N4	1.333 (4)	C9—C10	1.408 (6)
C1—C2	1.381 (5)	C9—H9	0.9300
C1—H1	0.9300	C10—C11	1.365 (5)
C2—C3	1.372 (5)	C10—H10	0.9300
C2—C6	1.504 (5)	C11—C12	1.397 (5)
C3—C4	1.376 (6)	C11—H11	0.9300
C3—H3	0.9300	C12—N1	1.356 (4)
C4—C5	1.364 (6)	N1—N2	1.321 (4)
C4—H4	0.9300	N1—Ag1^i^	2.311 (3)
C5—N4	1.329 (5)	N2—N3	1.324 (4)
C5—H5	0.9300	N5—O1	1.214 (4)
C6—N2	1.454 (4)	N5—O3	1.234 (4)
C6—H61	0.9700	N5—O2	1.236 (4)
C6—H62	0.9700		
			
N4—Ag1—N1^i^	131.66 (10)	C9—C8—H8	121.5
N4—Ag1—O3	127.43 (11)	C7—C8—H8	121.5
N1^i^—Ag1—O3	84.66 (11)	C8—C9—C10	122.0 (4)
N4—C1—C2	123.6 (3)	C8—C9—H9	119.0
N4—C1—H1	118.2	C10—C9—H9	119.0
C2—C1—H1	118.2	C11—C10—C9	122.4 (4)
C3—C2—C1	117.4 (3)	C11—C10—H10	118.8
C3—C2—C6	123.4 (3)	C9—C10—H10	118.8
C1—C2—C6	119.1 (3)	C10—C11—C12	116.2 (4)
C2—C3—C4	119.4 (4)	C10—C11—H11	121.9
C2—C3—H3	120.3	C12—C11—H11	121.9
C4—C3—H3	120.3	N1—C12—C11	130.6 (3)
C5—C4—C3	119.3 (4)	N1—C12—C7	107.9 (3)
C5—C4—H4	120.4	C11—C12—C7	121.5 (3)
C3—C4—H4	120.4	N2—N1—C12	103.1 (3)
N4—C5—C4	122.5 (4)	N2—N1—Ag1^i^	125.0 (2)
N4—C5—H5	118.8	C12—N1—Ag1^i^	129.0 (2)
C4—C5—H5	118.8	N1—N2—N3	117.4 (3)
N2—C6—C2	112.5 (3)	N1—N2—C6	121.5 (3)
N2—C6—H61	109.1	N3—N2—C6	121.1 (3)
C2—C6—H61	109.1	N2—N3—C7	102.5 (3)
N2—C6—H62	109.1	C5—N4—C1	117.8 (3)
C2—C6—H62	109.1	C5—N4—Ag1	119.5 (2)
H61—C6—H62	107.8	C1—N4—Ag1	122.6 (2)
N3—C7—C12	109.2 (3)	O1—N5—O3	120.7 (4)
N3—C7—C8	130.0 (4)	O1—N5—O2	119.0 (4)
C12—C7—C8	120.9 (3)	O3—N5—O2	120.3 (3)
C9—C8—C7	116.9 (4)	N5—O3—Ag1	111.5 (2)
			
N4—C1—C2—C3	2.0 (5)	C12—N1—N2—N3	−0.2 (4)
N4—C1—C2—C6	−176.4 (3)	Ag1^i^—N1—N2—N3	161.7 (2)
C1—C2—C3—C4	−0.4 (6)	C12—N1—N2—C6	−179.6 (3)
C6—C2—C3—C4	177.9 (4)	Ag1^i^—N1—N2—C6	−17.7 (4)
C2—C3—C4—C5	−1.2 (7)	C2—C6—N2—N1	74.0 (4)
C3—C4—C5—N4	1.6 (7)	C2—C6—N2—N3	−105.4 (4)
C3—C2—C6—N2	25.6 (5)	N1—N2—N3—C7	0.5 (4)
C1—C2—C6—N2	−156.1 (3)	C6—N2—N3—C7	179.9 (3)
N3—C7—C8—C9	−179.3 (4)	C12—C7—N3—N2	−0.7 (4)
C12—C7—C8—C9	0.3 (6)	C8—C7—N3—N2	179.0 (4)
C7—C8—C9—C10	−0.7 (6)	C4—C5—N4—C1	−0.1 (6)
C8—C9—C10—C11	0.5 (7)	C4—C5—N4—Ag1	−179.8 (3)
C9—C10—C11—C12	0.2 (6)	C2—C1—N4—C5	−1.7 (5)
C10—C11—C12—N1	178.7 (4)	C2—C1—N4—Ag1	177.9 (2)
C10—C11—C12—C7	−0.6 (5)	N1^i^—Ag1—N4—C5	−68.9 (3)
N3—C7—C12—N1	0.6 (4)	O3—Ag1—N4—C5	169.5 (3)
C8—C7—C12—N1	−179.0 (3)	N1^i^—Ag1—N4—C1	111.5 (3)
N3—C7—C12—C11	−179.9 (3)	O3—Ag1—N4—C1	−10.1 (3)
C8—C7—C12—C11	0.4 (5)	O1—N5—O3—Ag1	−1.8 (5)
C11—C12—N1—N2	−179.6 (4)	O2—N5—O3—Ag1	179.5 (3)
C7—C12—N1—N2	−0.3 (4)	N4—Ag1—O3—N5	−3.8 (3)
C11—C12—N1—Ag1^i^	19.5 (5)	N1^i^—Ag1—O3—N5	−144.1 (3)
C7—C12—N1—Ag1^i^	−161.1 (2)		

Symmetry codes: (i) −*x*+1, −*y*+1, −*z*+1; (ii) −*x*+1, *y*−1/2, −*z*+3/2.

### Hydrogen-bond geometry (Å, °)

**Table:**

*D*—H···*A*	*D*—H	H···*A*	*D*···*A*	*D*—H···*A*
C5—H5···O2^iii^	0.93	2.59	3.365 (3)	141
C6—H61···O2^iv^	0.97	2.48	3.416 (5)	161

Symmetry codes: (iii) *x*, −*y*+1/2, *z*−1/2; (iv) −*x*+1, *y*+1/2, −*z*+3/2.
